# Supplementary material for: The coiled-coil domain of Escherichia coli FtsLB is a structurally detuned element critical for modulating its activation in bacterial cell division
Source: J Biol Chem. 2021 Dec 4;298(1):101460. doi: 10.1016/j.jbc.2021.101460 (PMC8749076; doi:10.1016/j.jbc.2021.101460)
Supplement: Supplementary information [file mmc1.pdf]

SUPPLEMENTARY INFORMATION

The coiled-coil domain of *Escherichia coli* FtsLB is a structurally detuned element critical for modulating its activation in bacterial cell division

Samuel J. Craven[1,2], Samson G.F. Condon[1,2,], Gladys Díaz Vázquez[1,3], Qiang Cui[4], and Alessandro Senes[1,@]

[1]Department of Biochemistry, University of Wisconsin-Madison, Madison, WI 53706

[2]Integrated Program in Biochemistry, University of Wisconsin-Madison, Madison, WI 53706

[3]Biophysics Graduate Program, University of Wisconsin-Madison, Madison, WI 53706

[4]Department of Chemistry, Boston University

[@]Corresponding author: senes@wisc.edu

Supplementary tables

Table S1. Frequency of polar amino acids at *a* and *d* positions in a database of 2,662 crystal structures of coiled coils from the CC+ database.

Two-stranded parallel coiled coils			
Amino acid	*a* positions[1]	*d* positions[1]	*a*+*d* positions[1]
D	0.97%	1.13%	1.05%
E	3.34%	4.26%	3.80%
H	1.79%	1.46%	1.63%
K	4.72%	2.79%	3.76%
N	3.22%	1.72%	2.47%
Q	3.45%	2.57%	3.01%
R	4.47%	1.59%	3.03%
**Total polar**	**21.97%**	**15.53%**	**18.76%**

Three-stranded parallel coiled coils			
Amino acid	*a* positions[1]	*d* positions[1]	*a*+*d* positions[1]
D	0.38%	0.76%	0.57%
E	2.05%	1.39%	1.72%
H	1.15%	1.46%	1.31%
K	0.83%	0.95%	0.89%
N	1.86%	4.50%	3.19%
Q	5.00%	5.26%	5.13%
R	1.09%	1.46%	1.27%
**Total polar**	**12.38%**	**15.77%**	**14.09%**

Four-stranded parallel coiled coils			
Amino acid	*a* positions[1]	*d* positions[1]	*a*+*d* positions[1]
D	0.16%	1.20%	0.69%
E	2.86%	2.07%	2.46%
H	1.14%	1.59%	1.37%
K	1.14%	0.48%	0.81%
N	1.39%	1.75%	1.57%
Q	2.45%	5.18%	3.83%
R	0.49%	1.43%	0.97%
**Total polar**	**9.63%**	**13.71%**	**11.69%**

All parallel coiled coils			
Amino acid	*a* positions[1]	*d* positions[1]	*a*+*d* positions[1]
D	0.84%	1.08%	0.96%
E	3.14%	3.77%	3.46%
H	1.69%	1.45%	1.57%
K	4.00%	2.38%	3.19%
N	2.93%	1.99%	2.46%
Q	3.50%	3.10%	3.30%
R	3.77%	1.54%	2.65%
**Total polar**	**19.87%**	**15.32%**	**17.60%**

[1]Pseudocount-Adjusted frequency

Table S2. Alignment scores (Cα RMSD) of the five AlphaFold2 dimeric FtsLB models against half of the Y- and I-models ($FtsB_A$ 1-60, $FtsL_C$ 40-91)

Alphafold2 Model	pLDDT[1]	Y-model Cα RMSD (Å)	I-model Cα RMSD (Å)
**Rank_1**	79.78	2.33	3.47
**Rank_2**	78.12	2.26	3.40
**Rank_3**	77.63	2.27	3.36
**Rank_4**	76.35	2.35	3.26
**Rank_5**	61.94	2.71	2.31

[1]pLDDT score (predicted Local Distance Difference Test): Alphafold2's overall confidence metric of the model (100 = most confident, 0 = least confident)

Table S3. RMSD analysis of the three replica molecular dynamic runs of the FtsLB complex in the Y-model configuration

	RMSD average ± S.D. (Å)	Min RMSD (Å)	Max RMSD (Å)
**Run 1**			
Protein	8.3 ± 0.9	3.15	10.03
TM region	2.4 ± 0.4	1.41	2.98
Coiled coil domain, chain A and B	1.7 ± 0.4	0.85	2.94
Coiled coil domain, chain C and D	1.5 ± 0.3	0.82	2.43
Coiled coil, both domains	3.5 ± 0.6	1.55	5.16
Post-CCD, chain A and B	14.4 ± 1.8	2.46	17.00
Post-CCD, chain C and D	5.1 ± 0.7	2.74	6.76
**Run 2**			
Protein	7.7 ± 1.4	2.44	9.46
TM region	1.6 ± 0.3	0.92	2.05
Coiled coil domain, chain A and B	1.6 ± 0.3	0.89	2.35
Coiled coil domain, chain C and D	1.7 ± 0.3	0.88	2.57
Coiled coil, both domains	4.5 ± 0.7	1.63	6.14
Post-CCD, chain A and B	5.2 ± 1.8	1.27	11.69
Post-CCD, chain C and D	5.8 ± 1.5	1.99	7.88
**Run 3**			
Protein	7.5 ± 1.5	2.61	14.41
TM region	1.8 ± 0.2	1.24	2.32
Coiled coil domain, chain A and B	1.6 ± 0.3	0.89	2.35
Coiled coil domain, chain C and D	1.7 ± 0.3	0.88	2.57
Coiled coil, both domains	5.4 ± 1.0	2.06	10.65
Post-CCD, chain A and B	5.2 ± 1.8	1.27	11.69
Post-CCD, chain C and D	5.8 ± 1.5	1.99	7.88

Table S4. Strains and plasmids used in this work.

Strain/ plasmid	Description	Parent vector	Source
BL21(DE3)	chemically competent E. coli for protein overexpression	-	New England BioLabs (C2527)
NB946	FtsB depletion strain	-	Buddelmeijer et al., 2002[1]
MDG277	FtsL depletion strain	-	Gonzalez & Beckwith, 2009[2]
pSJC020	His-FtsB | Strep-FtsL35-121 C41A/C45A (Cys-less FtsL)	pETDuet-1	Condon et al., 2018[3]
pSJC309	His-FtsB Q39L/N43I | Strep-FtsL35-121 C41A/C45A/R67I/R74I	pETDuet-1	This paper
pNG162	IPTG-inducible, low-copy-number vector (empty)	pAM238	Goehring et al., 2006[4]
pMDG7	flag3-FtsB	pNG162	Gonzalez & Beckwith, 2009[2]
pSJC187	flag3-FtsB Q39L	pMDG7	This paper
pSJC188	flag3-FtsB N43I	pMDG7	This paper
pSJC208	flag3-FtsL N50I	pMDG7	This paper
pSJC287	flag3-FtsB A37D/A38D/A41D/A44E/A48E	pMDG7	This paper
pSJC288	flag3-FtsB A37E/A38E/A41E/A44D/A48D	pMDG7	This paper
pMDG29	flag3-FtsL	pNG162	Gonzalez et al., 2010[5]
pSJC183	flag3-FtsL R67I	pMDG29	This paper
pSJC185	flag3-FtsL R74I	pMDG29	This paper
pSJC190	flag3-FtsL R67I/R74I	pMDG29	This paper
pSJC194	flag3-FtsL R67E	pMDG29	This paper
pSJC201	flag3-FtsL R74E	pMDG29	This paper
pSJC220	flag3-FtsL W81I	pMDG29	This paper
pSJC254	flag3-FtsL R67E/R74E	pMDG29	This paper
pSJC304	flag3-FtsL R67I/R74E	pMDG29	This paper
pSJC324	flag3-FtsL R67K	pMDG29	This paper
pSJC325	flag3-FtsL R74K	pMDG29	This paper
pSJC326	flag3-FtsL R67E/R74I	pMDG29	This paper
pSJC334	flag3-FtsL R74E/W81I	pMDG29	This paper
pSJC335	flag3-FtsL R74K/W81I	pMDG29	This paper
pSJC336	flag3-FtsL R67I/R74K	pMDG29	This paper
pSJC337	flag3-FtsL R67E/R74K	pMDG29	This paper

Supplementary references

(1) Buddelmeijer, N., Judson, N., Boyd, D., Mekalanos, J. J., and Beckwith, J. (2002) YgbQ, a cell division protein in Escherichia coli and Vibrio cholerae, localizes in codependent fashion with FtsL to the division site. *Proc. Natl. Acad. Sci. U. S. A. 99*, 6316–6321.
(2) Gonzalez, M. D., and Beckwith, J. (2009) Divisome under construction: distinct domains of the small membrane protein FtsB are necessary for interaction with multiple cell division proteins. *J. Bacteriol. 191*, 2815–2825.
(3) Condon, S. G. F., Mahbuba, D.-A., Armstrong, C. R., Diaz-Vazquez, G., Craven, S. J., LaPointe, L. M., Khadria, A. S., Chadda, R., Crooks, J. A., Rangarajan, N., Weibel, D. B., Hoskins, A. A., Robertson, J. L., Cui, Q., and Senes, A. (2018) The FtsLB subcomplex of the bacterial divisome is a tetramer with an uninterrupted FtsL helix linking the transmembrane and periplasmic regions. *J. Biol. Chem. 293*, 1623–1641.
(4) Goehring, N. W., Gonzalez, M. D., and Beckwith, J. (2006) Premature targeting of cell division proteins to midcell reveals hierarchies of protein interactions involved in divisome assembly. *Mol. Microbiol. 61*, 33–45.
(5) Gonzalez, M. D., Akbay, E. A., Boyd, D., and Beckwith, J. (2010) Multiple interaction domains in FtsL, a protein component of the widely conserved bacterial FtsLBQ cell division complex. *J. Bacteriol. 192*, 2757–2768.

Supplementary figures

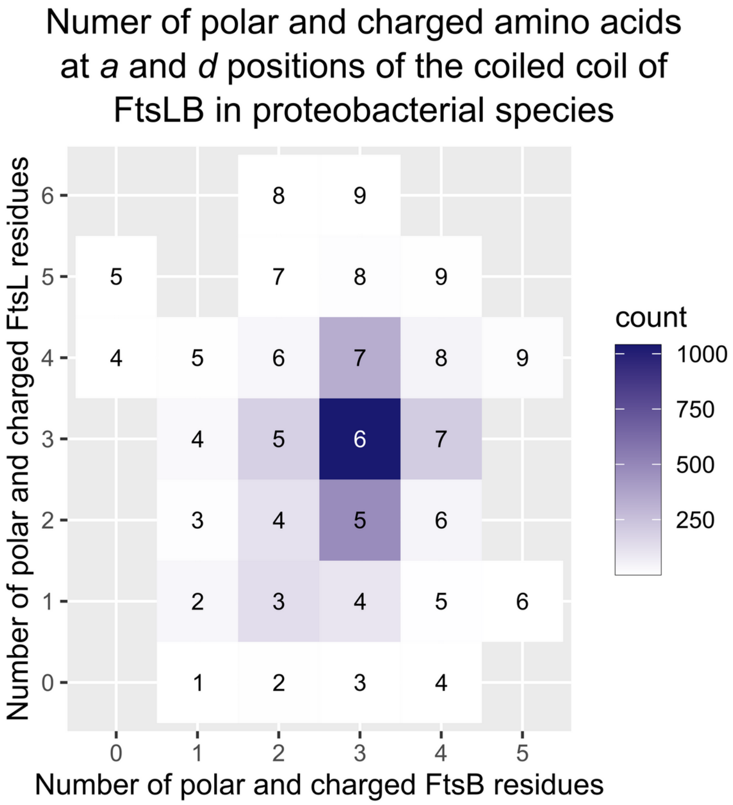

Fig. S1. Conservation of the polar cluster in the core "*a*" and "*d*" positions of the coiled coil of FtsLB in proteobacterial species. Number of sequences with a given number of polar/charged amino acids (Asp, Glu, His, Arg, Lys, Gln, and Asn) contributed by FtsB (X axis) and FtsL (Y axis) at "*a*" and "*d*" positions in the five heptad repeats of the coiled coil (their sum is reported in the box). Data from an alignment of 2900 paired FtsB/FtsL proteobacterial sequences. The most frequent combination corresponds, by a large margin, to three polar residues contributed by both FtsB and FtsL, for a total of six polar residues. Although less frequent, combinations with a total of five or seven polar residues also occur relatively often. Overall, 84% of the sequences contain at least 5 polar residues.

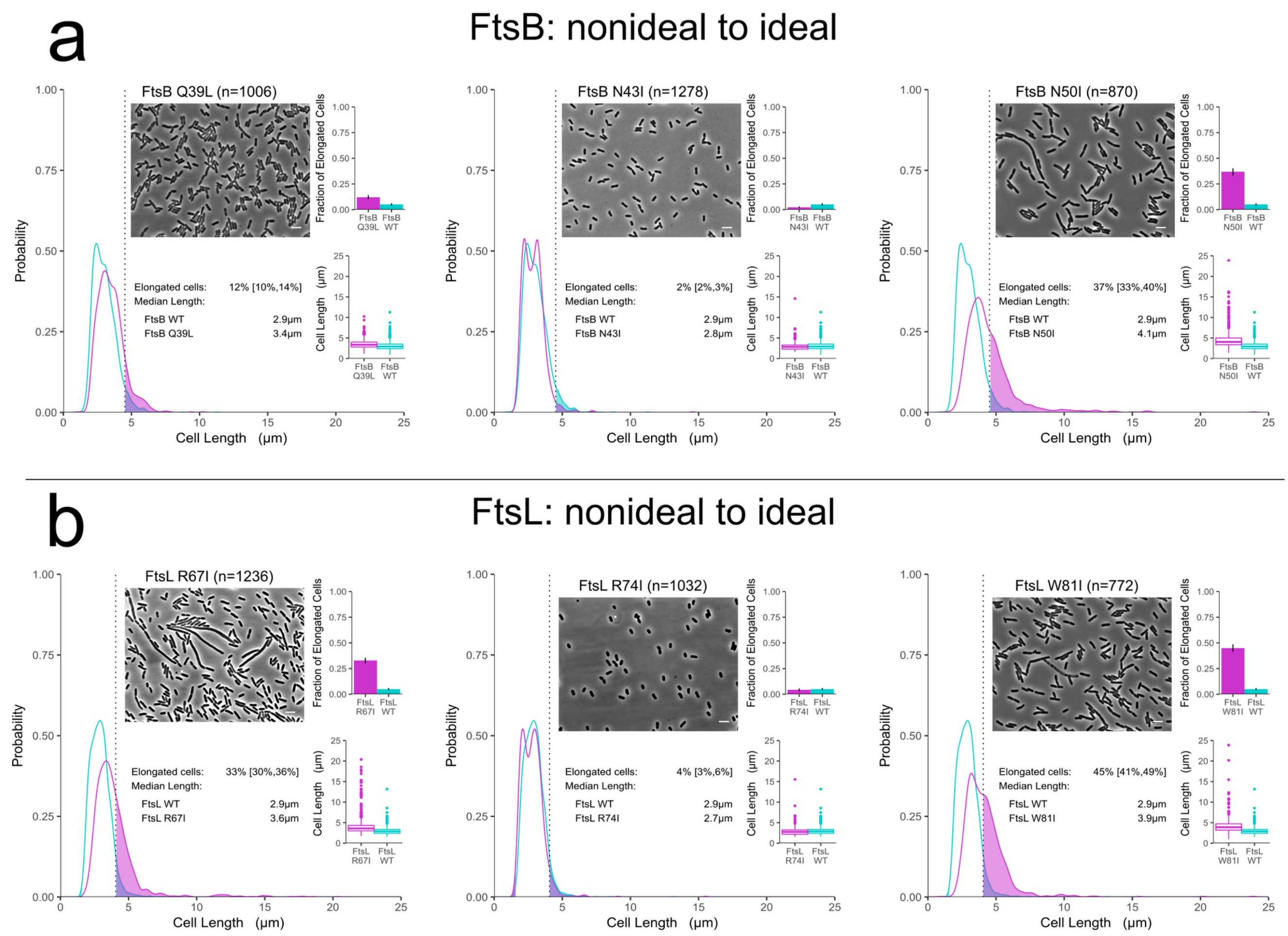

Fig. S2. Cell length distribution of mutants. Phase-contrast images and cell length distributions of mutant cells (magenta) compared to wild-type (cyan). White scale bar = 5 µm. The shaded areas to the right of the dotted lines represent the fraction of cells that are longer than the 95th percentile in the WT distribution. The fraction is plotted as a histogram in the top inset. Error bars and bracketed values represent the 95% confidence interval for the fraction of elongated cells, estimated by 1000 bootstrap replications of the samples. The bottom inset is a box and whisker plot of the same distribution of the main panel. *a*) Nonideal to ideal mutations in FtsB. *b*) Nonideal to ideal mutations in FtsL. Next pages: *c*) Charge variations in FtsL at R67 and R74 positions. *d*) Double substitutions in FtsL at R67, R74, and W81 positions. *e*) Ala patch substitutions in FtsB in which all five Ala positions (37, 38, 41, 44, and 48) were simultaneously replaced with negatively charged residues, with a combination of three Glu and two Asp residues (EEEDD) and vice versa (DDDEE). Experiments were performed at 37 °C.

(continued on next page)

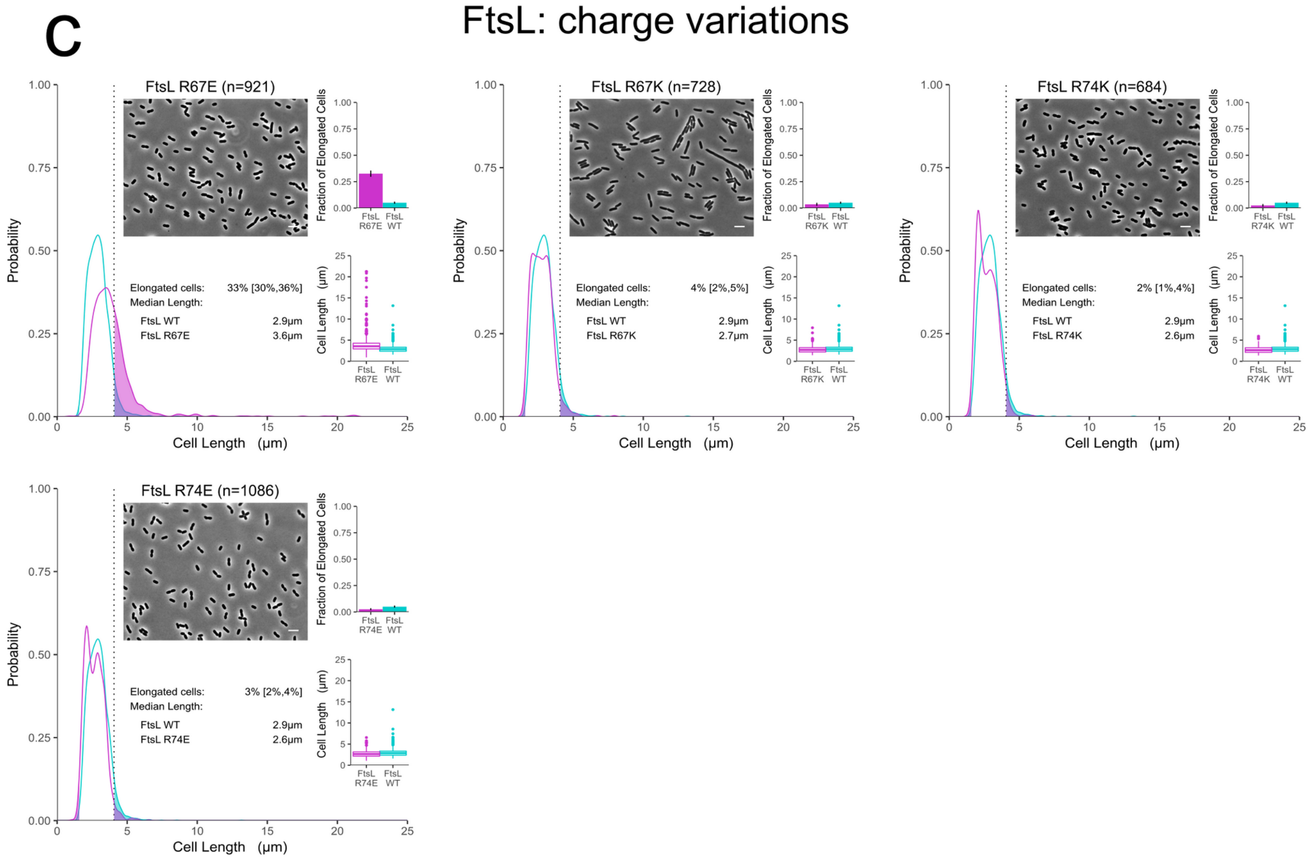

Fig. S2 (continued from previous page and continues on next page).

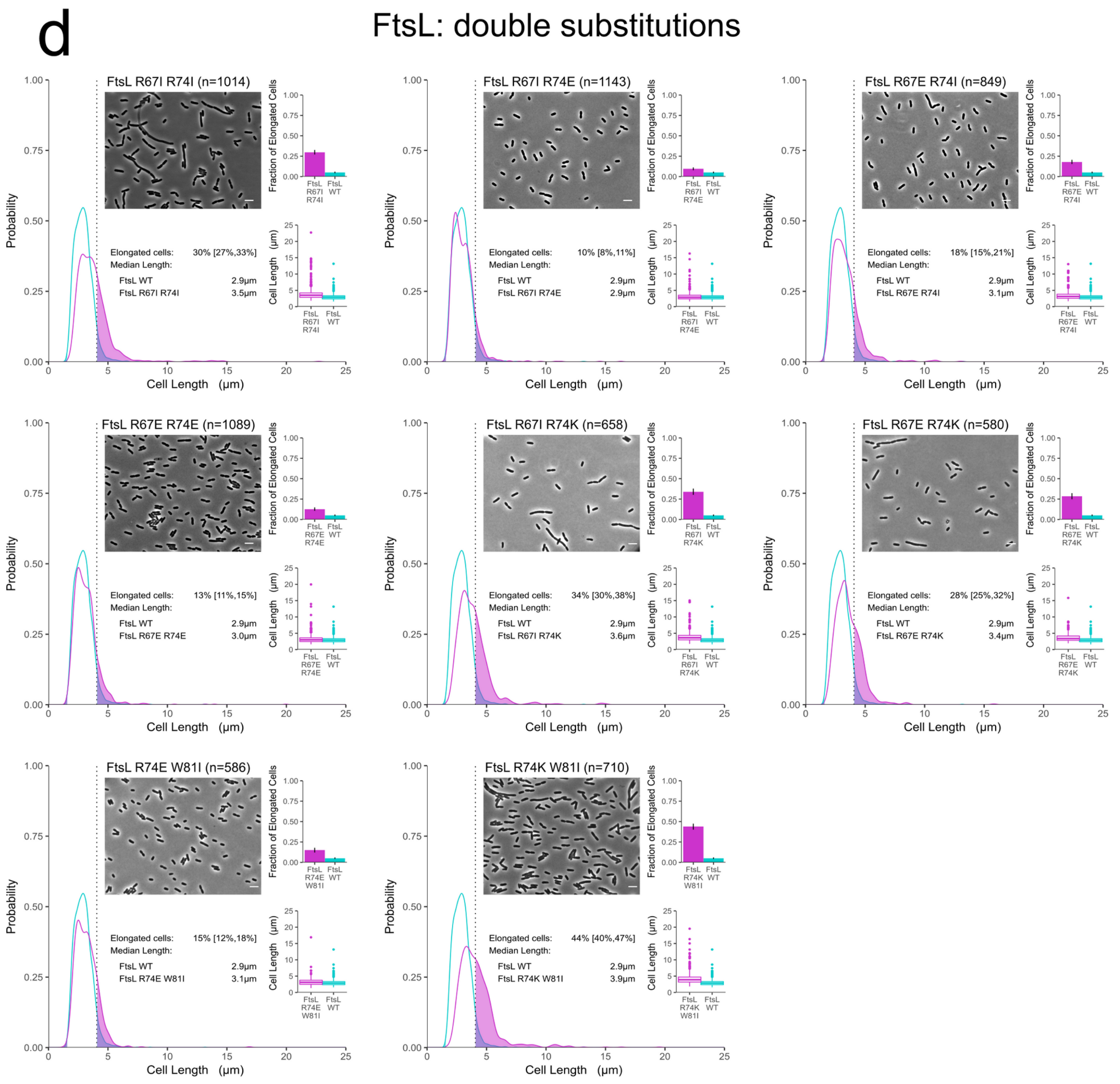

Fig. S2 (continued from previous page and continues on next page).

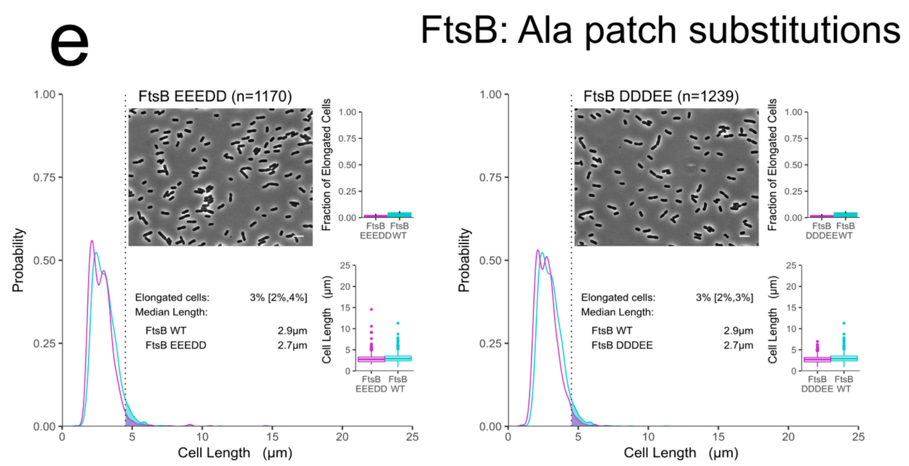

Fig. S2 (continued from previous page).

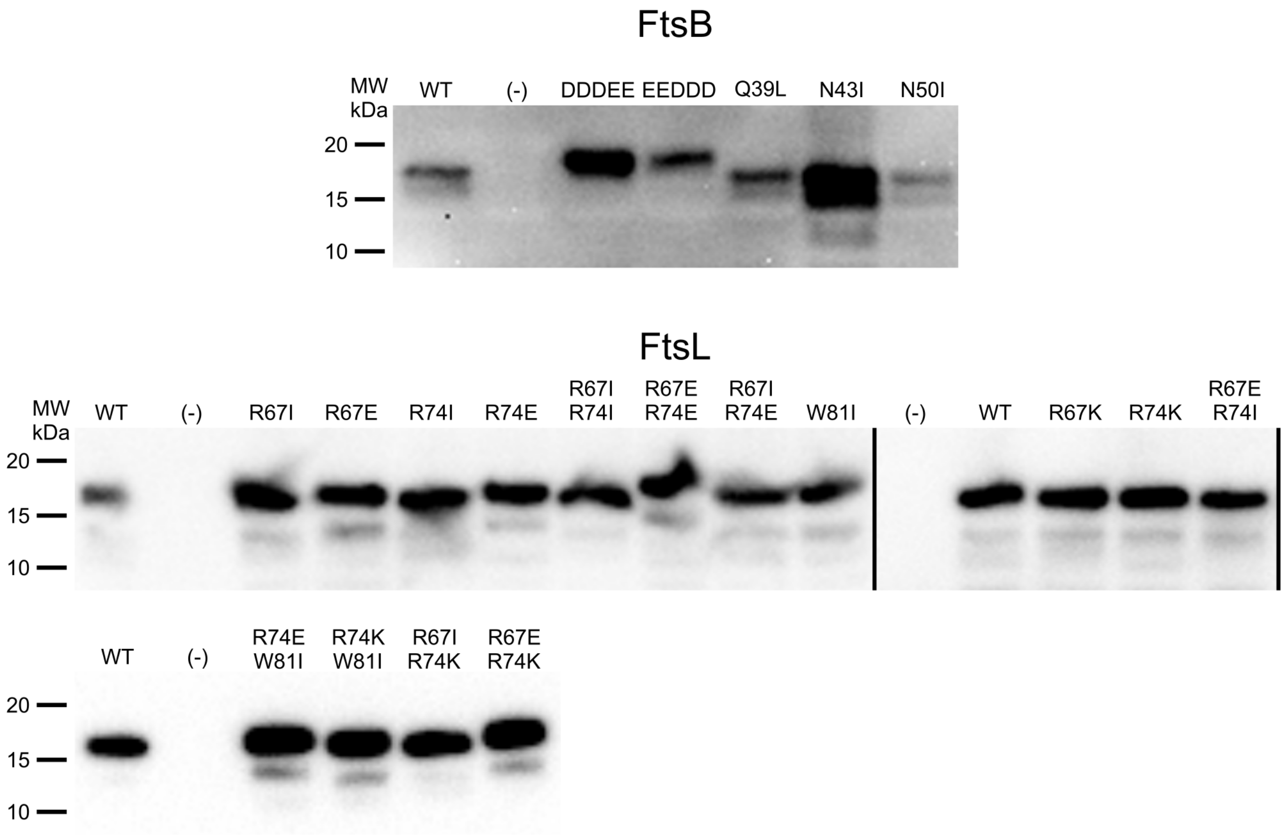

Fig. S3. Expression level of FtsB and FtsL mutants assessed by Western Blot analysis. Image of all western blots of the FtsB and FtsL mutants tested in this work. Around twice as much whole cell lysate (normalized to total protein) was loaded for FtsB samples as for FtsL samples. Protein expression level of the FtsB and FtsL mutants with defective phenotypes are generally comparable to the respective wild type (WT). Negative controls (-) show no detectable signal for either protein. DDDEE is FtsB A37D/A38D/A41D/A44E/A48E, and EEEDD is FtsB A37E/A38E/A41E/A44D/A48D. Both show a slightly increased molecular weight, which may be due to the increased number of negatively charged residues in these mutants. There are cases of FtsB mutants with increased protein level (N43I, in particular), though it is unclear why. Individual gels are separated by solid lines.

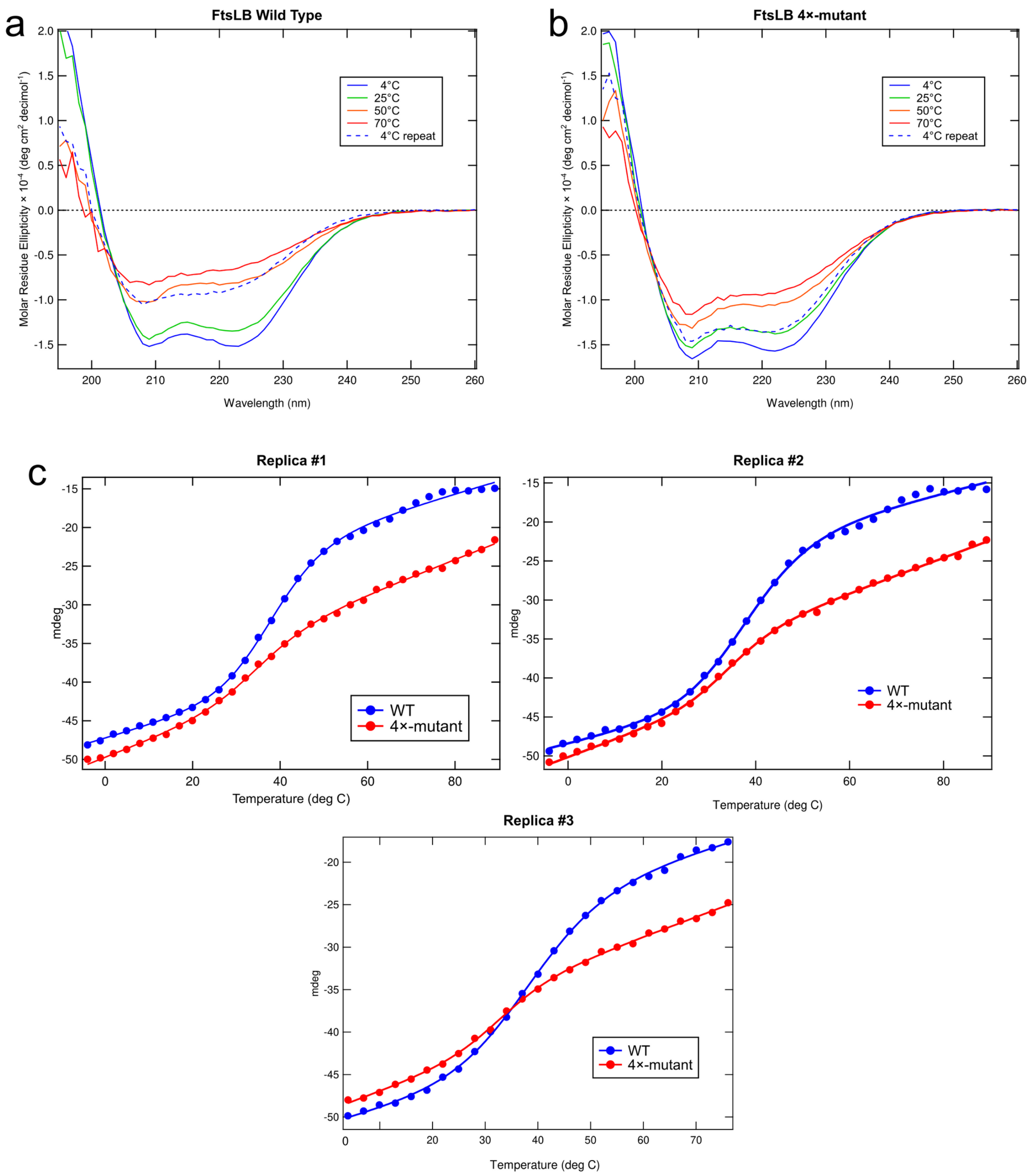

Fig. S4. a) Far-UV CD spectra of WT FtsLB at 4, 25, 50, and 70 °C along with a 4 °C repeat after cooling the sample. b) Same analysis of the 4×-mutant. c) Replica experiments of temperature melting curves comparing WT FtsLB (blue) to the 4×-mutant (red). CD scans were monitored at 224 nm from 4 °C to 89 °C.

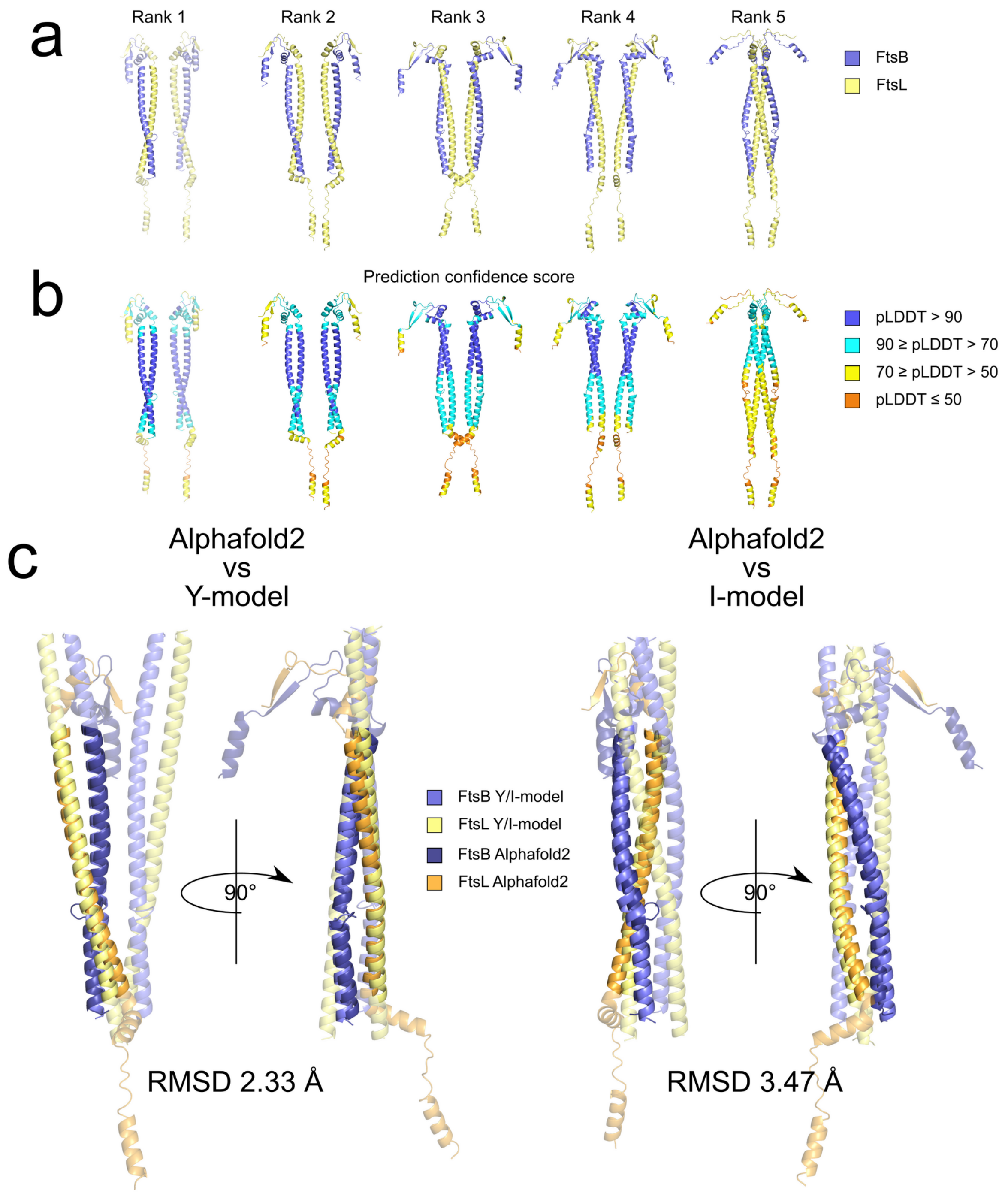

Fig. S5. Alphafold2 models of FtsLB. a) Alphafold2 produced five ranked models of FtsLB, numbered 1-5 (best to worst). In spite of four subunits being provided, rank models 1-4 display separated dimeric units with little or no interaction between them. Rank model 5 is organized in a tetrameric unit. However, the interactions between the pair of dimers are loose and significantly underpacked. b) The Alphafold2 models have high confidence in the coiled-coil region, as color coded in the figure (100: highest confidence; 0: lowest confidence). c) Alignments of one dimer from the rank 1 model to half of the Y-model (left) and the I-model (right). Regions not used in the alignment are transparent. The alignment with the Y-model is excellent, with a Cα RMSD of 2.33 Å, while the I-model alignment is less optimal, with an RMSD of 3.47 Å.

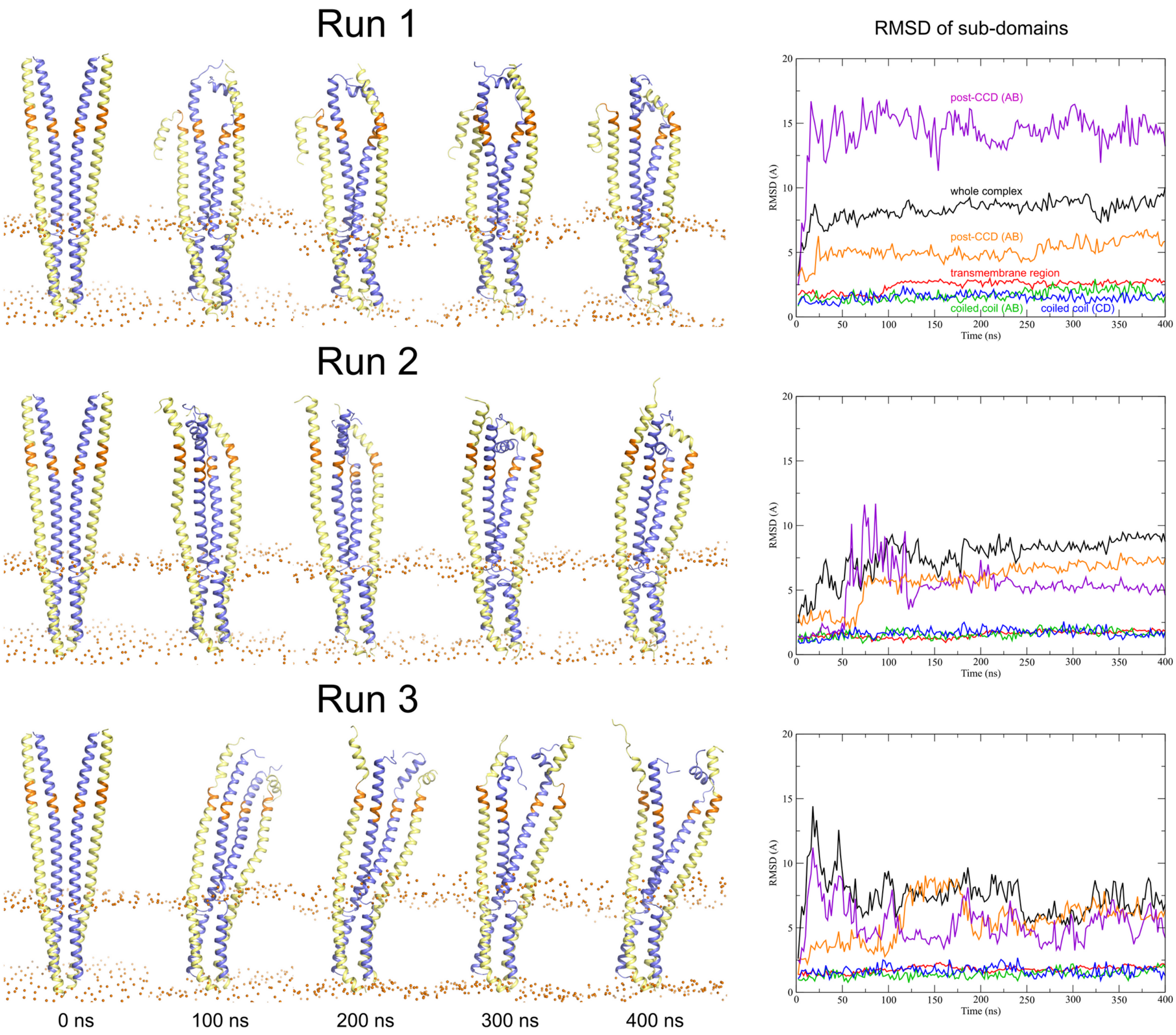

Fig. S6. Trajectories of the three 400 ns replica MD runs of the FtsLB Y-model. The graph illustrates the fluctuations of the RMSD of the entire complex (black) and the individual subdomains: red: transmembrane region; green: coiled coil, chains A (FtsB) and B (FtsL); blue: coiled coil, chains C (FtsB) and D (FtsL); magenta: post-CCD region, chains A and B; orange: post-CCD region, chains C and D. The RMSD indicates that the transmembrane domain and coiled-coil domains remain relatively stable during the entirety of the simulations. The RMSD analysis is summarized in supplementary Table S3.
